# In silico approach in reveal traditional medicine plants pharmacological material basis

**DOI:** 10.1186/s13020-018-0190-0

**Published:** 2018-06-19

**Authors:** Fan Yi, Li Li, Li-jia Xu, Hong Meng, Yin-mao Dong, Hai-bo Liu, Pei-gen Xiao

**Affiliations:** 1 Key Laboratory of Cosmetic, China National Light Industry, Beijing Technology and Business University, No. 11/33, Fucheng Road, Haidian District, Beijing, 100048 People’s Republic of China; 2 Beijing Key Laboratory of Plant Resources Research and Development, Beijing Technology and Business University, No. 11/33, Fucheng Road, Haidian District, Beijing, 100048 People’s Republic of China; 3 Institute of Medicinal Plant Development, Chinese Academy of Medical Sciences, Peking Union Medical College, Beijing, 151 Malianwa North Road, Haidian District, Beijing, 100193 People’s Republic of China

**Keywords:** Traditional medicinal plants, Virtual screening, Network pharmacology, Medicinal plants information analytics, Pharmacological basis

## Abstract

In recent years, studies of traditional medicinal plants have gradually increased worldwide because the natural sources and variety of such plants allow them to complement modern pharmacological approaches. As computer technology has developed, in silico approaches such as virtual screening and network analysis have been widely utilized in efforts to elucidate the pharmacological basis of the functions of traditional medicinal plants. In the process of new drug discovery, the application of virtual screening and network pharmacology can enrich active compounds among the candidates and adequately indicate the mechanism of action of medicinal plants, reducing the cost and increasing the efficiency of the whole procedure. In this review, we first provide a detailed research routine for examining traditional medicinal plants by in silico techniques and elaborate on their theoretical principles. We also survey common databases, software programs and website tools that can be used for virtual screening and pharmacological network construction. Furthermore, we conclude with a simple example that illustrates the whole methodology, and we present perspectives on the development and application of this in silico methodology to reveal the pharmacological basis of the effects of traditional medicinal plants.

## Background

Over three quarters of the world’s population relies mainly on plants and plant extracts for health care. World Health Organization (WHO) report indicated that over 30% of all plant species have at one time or another been used for medicinal purposes [1]. The scientific study of traditional medicinal plants is of great importance for human health.

Identifying and predicting the pharmacological basis of the activity of traditional medicinal plants are important for the goal of modernizing their use [2]. Because the chemical constituents of medicinal plants are complex and varied, clarifying the specific chemical components in such plants and their major biological functions is a complex task.

The traditional model of medicinal plant research can usually be divided into the following steps: first, the extraction of compound monomers or fractions, followed by their qualitative and quantitative identification and then a variety of pharmacological experiments such as in vitro experiments and injection or feeding these solution in animal then performing effective measurement [3]. In general, the whole research process is time consuming and expensive. However, several parts of this common approach can be modified to improve the efficiency. At present, the chemistry of the most widely used medicinal plants, such as *ginseng* and *licorice*, has been studied quite thoroughly. Hundreds of chemical compounds have been extracted from these medicinal herbs. However, most of these compounds have not to be studied whether they have potential no biological activity. It would be a vast and time-consuming project to systematically evaluate the activities of these ingredients by conventional methods.

In recent years, with an increasingly in-depth understanding of the structure and function of compounds, a series of new technologies and methods have been applied to the development of medicinal plants [4]. If we can establish a quick and convenient pathway by which to first accurately predict a large number of chemical compounds and then, based on these results, perform in vivo and in vitro pharmacological experiments for verification, this procedure will significantly improve the efficiency of evaluating the chemical activities of medicinal plants [5]. Back to 1950s, Artemisinin, the most famous antimalarial drug, was also obtained through large-scale screening from herbs. After that, several FDA approved drugs were developed from natural herbs or animal through the in silico approaches. The first case use enzyme-inhibitor structure to develop drug was the Angiotensin converting enzyme inhibitor-Captopril in 1970s [6]. And the first FDA approved HIV-1 enzyme inhibitor—Saquinavir also developed by in silico approaches [7]. Nowadays, with the continuous advancement of computer science, successful examples of finding drugs from natural products using computer-aided drug design methods have become more frequently, such as Dozamide (Approved by FDA in 1995), Imatinib (Approved by FDA in 2001), Dasatinib (Approved by FDA in 2006), and Ponatinib (Approved by FDA in 2012) [8]. With the continuous maturation of computer technology, the in silico approach of utilizing a computer platform to calculate the combinations of simulated compounds and targets has become increasingly accurate. In addition, the development of network pharmacology technologies has enabled the rapid elucidation of the complex relationships between compounds and their various activity targets [9]. Traditional Chinese medicine (TCM) involves a highly complex system of chemical substances. Its complexity is not only reflected in the composition of the chemical constituents but also embodied in the network of relationships between the prescription and the human body and the exertion of pharmacological effects through multiple channels, multiple targets and the overall regulatory mechanism [10].

**Fig. 1:**
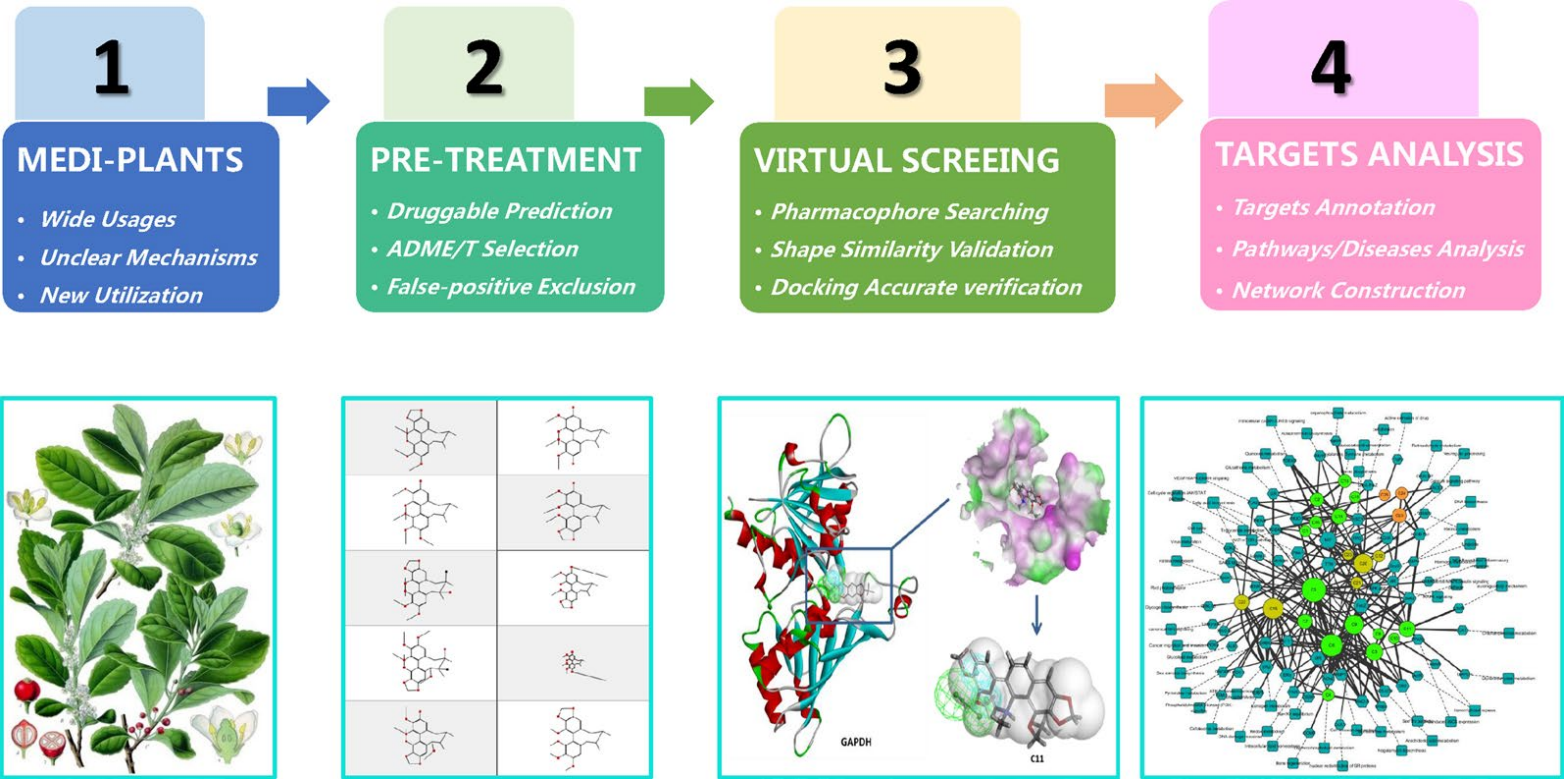
The overall design of the methodology

Regarding the implications of Chinese medicine informatics, the application of modern informational technology to study TCM must address information on a variety of characteristics of the medicines themselves, as well as the interactions between TCMs and the human body [11]. The definition of Chinese medicine informatics is as follows: an interdisciplinary science that applies information science theory and computer technology to study the regulation and process of TCM information flow and investigates the views of TCM practitioners for the purpose. The definition of network pharmacology is as follows: a new discipline based on systems biology theory, in which the network analysis of biological systems is used to select the characteristics of signal nodes for multi-target small molecular drug design [12]. Therefore, we can treat a single medicinal plant with the same complexity as TCM, utilizing technical means from TCM informatics and network pharmacology to explore the chemical composition and the potential pharmacological foundation of the plant’s effects. In this paper, we elucidate the methodology in detail (Fig. 1).

## Design of the methodology

In this paper, we elucidate the methodology in detail (Fig. 1). Considering the medicinal plant as the object and virtual computational screening as the central methodology within Chinese medicine informatics, and using network pharmacology as the technical means, we have employed a set of effective and accurate methods to reveal the pharmacological basis of the effects of medicinal plant materials and predict the potential bioactivity of their compounds. The approach proceeds from the selection of the plant compounds to virtual screening and evaluation of the targets, related signalling pathways and disease networks. Techniques from virtual computational screening, Chinese medicine informatics and network pharmacology are used to construct a complete technical routine for this methodology. The first step is to confirm the research significance of the plants. These medicinal plants can be organized into three categories: (1) common herbs with a more complex mechanism than that of other herbs; (2) herbs with a long history of traditional usage but fewer studies on their phytochemistry and pharmacology; and (3) herbs with a history of traditional usage but now with a new utilization. The second step is the organization and collection of the natural products to construct the natural products database. The third step is the pre-treatment of these compounds, including drug-like analysis, ADME/T (absorption, distribution, metabolism, excretion and toxicity) prediction and the exclusion of false-positive compounds. The fourth step is the core technique of this methodology, the in silico virtual screening. We designed this step by combining three different theoretical bases: the first is virtual target fishing based on pharmacophore theory; the second is dual validation based on small molecule shape similarity theory; and the third is compound-target analysis based on docking. The fifth step is the analysis of the set of targets identified. By utilizing the technical methods of network pharmacology and different protein information databases and websites, we can analyse the relationships of signalling pathways, pathological pathways and related diseases with potential targets. Finally, we construct the network of relationships among medicinal plants, natural compounds, biological targets, signalling pathways, and diseases. We thereby elucidate the mechanistic basis of the effects of the natural compounds in medicinal plants and predict their potential pharmacological activities.

### Confirmation of the objective

At present, the pharmacological mechanisms of numerous single compounds extracted from herbs have been elucidated. However, herbs contain diverse and complex compounds that are involved in multiple biological pathways and that correspond to different diseases. Therefore, investigating the different types of medicinal plants and their chemical constituents is a task with both broad and specific significance. We have recommended three categories of herbs that are suitable for the use of the in silico approach to unveil their chemical mechanisms. The first type of medicinal plants can be described by “common usage, but their mechanism is more complicated”, such as *Panax notoginseng* (Burk.) F. H. Chen (Notoginseng), which is mainly cultivated in Yunan, China [13]. The medicinal part of notoginseng is its dry roots, and their main clinical activity is “scattered blood stasis, swelling pain”. The noteworthy point is that notoginseng can not only promote blood circulation but also stop bleeding. The common theory regarding this two-way regulatory function is that the notoginseng saponins can invigorate the circulation of blood, while dencichine performs a haemostatic function [14]. Therefore, it is necessary to identify the pharmacological basis of the effects of the constituents of these herbs. The second type of medicinal plants is ones that “have a long history of traditional usage, but their study is in the early stages and not yet mature”. Several medicinal plants in different regions have a history of more than 1000 years of traditional usage, such as “Peruvian ginseng” *Lepidium meyenii* Walp (maca), which is mainly distributed in the Andean mountains in the south of Peru at an altitude greater than 3000 m. The local people have consumed the subterranean part of maca to enhance their energy, improve their fertility and sexual function, or treat menopausal syndromes, rheumatism, depression, and anaemia for more than 1000 year [15]. However, pharmacological research on such plants only began in the last decade, so research on these medicinal plants is particularly important and urgent. The third category of medicinal plants are those that have new and different uses. To shorten the development cycle of new drugs, reduce risk and improve the success rate, new uses of traditional herbs in other diseases are often proposed. Berberine, also known as puerarin, is mainly extracted from *Coptis chinensis* Franch and has anti-bacterial activity. In vitro experiments show broad-spectrum anti-bacterial activity, which is mainly used clinically to treat digestive system infections such as acute gastroenteritis and dysentery. Interestingly, in recent years, researchers have found that berberine has an excellent effect in the treatment of cardiovascular disease [16]. Artemisinin, extracted from *Artemisia annua* L., was the most efficient anti-malarial drug in use for decades. Researchers have recently found that it can also counteract tuberculosis, diabetes, and trauma, treat systemic lupus erythaematosus and perform other pharmacological functions [17].

Research on the development and utilization of three types of medicinal plants is particularly likely to be valuable. Once the objectives (medicinal plants) have been selected for study, we can perform the next step, the collection of their compounds.

### The acquisition of chemical compound information

A thorough understanding of the effective compounds in medicinal plants is the key to the research and development of medicinal plants. Therefore, the collection of constituent information and the construction of the compound database are highly important for their application. The construction of a compound database can effectively manage the large quantities of compounds found in medicinal plants.

#### Collection of chemical compound information

The information contained in a medicinal plant is the initial raw material for determining the basis of the herb’s pharmacological properties. Compound information was mainly collected from the following sources: (1) separation and purification of the compounds in a local laboratory; (2) literature reports; and (3) small molecule compound databases. Among these three information-gathering pathways, the extraction of compounds in a local laboratory is the most direct and convenient method and can provide samples for later experimental studies. When a single compound is purified from herbs, the relevant information such as its recording number, CAS number, name, source plant, extractive fraction and structure information such as the SMILES code or InChiKey must be recorded. The compounds obtained from the laboratory are doubtless the best research objects; however, these compounds are often either relatively simple or difficult to obtain intact as constituents of a given herb. Therefore, the literature and various databases may offer a simple way to collect information on a set of different compounds from our herb of interest. To use these resources, relevant information such as the name, structure, classification and plant source must be recorded. With the continuous upgrades to virtual screening technology, the online large-scale direct search database has become a quicker and more convenient approach. At present, various research institutions, laboratories, government agencies and pharmaceutical enterprises have developed and constructed a number of small molecule compound databases reporting different characteristics and functions (Table 1).

**Table 1:** Common small molecular databases overview

Databases	Development organizations	Websites	Overview	References
Alkamid Database	Ghent University, Belgium	http://www.alkamid.ugent.be	Provided the source of plants, biosynthetic pathways and pharmacological information of *N*-alkyl amides compounds in traditional medicinal plants	[18]
Asian Anti-Cancer Materia Database	Institute of East-West Medicine, USA	http://www.asiancancerherb.info/herbList/list_s.htm	Summarizes 700 kinds of anti-cancer drug information from Asia, which 80% is derived from medicinal plants. Afford the commonly used Chinese medicine name, Latin name, medicinal properties, the major compounds and other information	[19]
Chem-TCM	Institute of Pharmaceutical Science at King’s College, UK	http://www.chemtcm.com	Contains more than 350 TCMs and their more than 9500 compounds. Record their compounds related plants, chemical properties, common target activities and other information	[20]
Chinese National Compound Library	National Health and Family Planning Commission of the People’s Republic of China	http://www.app.cncl.org.cn	A library of small molecule compounds consisting of core libraries and satellite libraries. Contains nearly 2 million small molecules of the physical and chemical information	[20]
CHMIS-C (A Comprehensive Herbal Medicine Information System for Cancer)	University of Michigan Medical School, USA	http://www.sw16.im.med.umich.edu/chmis-c	Provided 527 anti-cancer herb prescriptions, 937 components and 9366 small molecule structures for the clinical treatment of different types of cancer, combined a reference database and a molecular target aided database	[21]
CNPD (Chinese Natural Products Database)	Shanghai Institute of Materia Medica, Chinese Academy of Sciences, China	http://www.neotrident.com/product/detail.aspx?id=16	The CNPD database currently collects more than 57,000 natural products from 37 categories, of which 70% of the molecules are drug-like molecules. The relevant data include the CAS number, name, molecular formula, molecular weight, melting point and other physical and chemical properties of natural products	[21]
KEGG Compound Database	Kyoto University, Japan	http://www.genome.jp/kegg/compound	Contains the name, molecular formula, relative molecular mass, structural formula, CAS number and corresponding chemical reaction or metabolic pathway of more than 17,000 metabolites and other small molecule compounds in the KEGG database	[22]
NAPRALERT (Natural Products Alert)	College of Pharmacy University of Illinois at Chicago, USA	http://www.napralert.org	A natural product relationship database according to more than 200,000 references which including pharmacology, biochemical information and data in various experiments (in vivo, in vitro, clinical, etc.)	[23]
NCI	National Cancer Institute, USA	https://cactus.nci.nih.gov/download/nci/	Database contains the chemical properties of compounds, such as the molecular formula, CAS number, and other common physical and chemical properties; anti-HIV activity and other related biological activity prediction value	[24]
TCM Database@Taiwan	China Medical University, Taiwan	http://www.tcm.cmu.edu.tw	Including 443 Traditional Chinese medicine and 20,000 kinds of ingredients, for the physical and chemical properties and 3D structure. Support the complete contents of the compounds from Chinese medicine and related references	[25]
TCMID (Traditional Chinese medicine Integrated Database	East China Normal University, China	http://www.megabionet.org/tcmid	Provide information on all aspects of Chinese medicine, including formula, herbal and herbal ingredients. Also collected information on drugs that are studied in depth by modern pharmacology and biomedical science	[26]
TCMSP	Center for Bioinformatics, College of Life Science, Northwest A&F University, China	http://www.lsp.nwsuaf.edu.cn/tcmsp.php	Contained 499 Chinese Parmacopoeia recorded TCMs and 29,384 compounds from these TCMs	[27]
Timtec	Timtec, Russia	http://www.timtec.net	Record more than 13,000 natural products and their derivatives structure information	[28]
TradiMed (Traditional Chinese medicine DB)	TradiMed, Korea	http://www.tradimed.com	Contains 11,810 prescriptions in 3199 Chinese and Korean traditional medicines and 20,012 chemical composition information	[29]
ZINC	University of California, USA	http://www.zinc.docking.org	Free compounds virtual screening database which included more than 35 million kinds of commercially available compounds 3D structure for docking	[30]

#### Software and database of medicinal plant compounds

Many commercial or access-restricted software programs or websites can satisfy the needs of constructing a medicinal plant compound database. Different software programs focus on specific functions embodied in diverse storage formats, for instance, the accdb format of Microsoft Company, the MDB format within the MOE software, and the SDF format from Accelary Discovery Studio. A complete medicinal plant compound database must provide at least the following descriptors: (1) compound storage number; (2) compound name and CAS (Pubchem) ID; (3) sources of plant information (Latin name and extractive fraction); and (4) compound structure (SMILES code, InChiKey). In addition, several software programs can also calculate the relative molecular weight, the lipid partition coefficient (AlogP), the number of rotatable bonds, the number of hydrogen donors/receptors and other physical and chemical properties of compounds. The combination of all this relatively trivial information can make follow-up work requiring transparency and data processing more convenient.

### Pre-treatment of chemical compounds

The number of compounds collected from medicinal plants is very high; however, the majority lack pharmacological potency. To enhance the efficiency of screening, the first step is to remove these non-potential compounds and refine the included compounds.

#### Prediction of drug-like properties

Drug-like characteristics are a qualitative concept used in drug design for a compound’s utility with respect to factors such as bioavailability, which is estimated based on the molecular structure characteristics [31]. Certain structure properties indicate that a compound has a higher likelihood of becoming a successful drug. In the past, research on these properties of a drug has been among the most important components of downstream drug development. In recent years, it has become imperative to integrate the study of drug properties during the early stages of drug discovery. Pharmacologists are interested in the following properties of the drugs, among others: (1) structural characteristics: hydrogen bonding, polar surface area, lipophilicity, shape, molecular weight, and acid dissociation constant (*pKa*); (2) physicochemical properties: solubility, pH value, permeability and chemical stability; (3) biochemical properties: metabolism, protein binding affinity and transport ability; and (4) pharmacokinetics and toxicity: half-life, bioavailability, drug interactions and half lethal dose, LD50. According to Lipinski’s proposal [32], a small molecule suitable for development as a drug needs the following properties (Lipinski’s rule of five, RO5): (1) no more than 5 hydrogen bond donors (the total number of nitrogen–hydrogen and oxygen–hydrogen bonds); (2) no more than 10 hydrogen bond acceptors (all nitrogen or oxygen atoms); (3) a molecular mass less than 500 Daltons; and (4) an octanol–water partition coefficient logP not greater than (5) Small molecules that satisfy the RO5 criteria have higher bioavailability in the metabolic process of the organism and therefore are more likely to become oral medications. In 2002, Veber and his group presented another set of system requirements for the oral administration of drugs that included the molecular flexibility, polar surface area (PSA) and number of hydrogen bonds, which were determined by a series of studies in rats: (1) no more than 10 rotatable bonds and (2) polar surface area no more than 140 Å^2^ or no more than 12 hydrogen bonds and receptors [33]. However, there are still exceptions for special drug screening such as narcotic drug screening, which cannot rule out small molecular weight compounds, and those anti-tumour drugs cannot exclude metal organic compounds. Nowadays, several softwares can performing the drug-likeness prediction. Such as the Instant JChem from ChemAxon company which can calculate the RO5 properties, and the Discovery Studio software can both predict the Lipinski and Veber’s rules [34].

#### ADME/T selection

When drug-likeness established from the analyses of the physiochemical properties and structural features of existing drug candidates, the ADME/T (absorption, distribution, metabolism, excretion and toxicity) properties play an important role in the drug filtering. So, we employed the ADME/T selection after other drug-likeness properties evaluated.

It is necessary to predict the situation and movement of a drug in the human body during the design of the drug molecule. During the early stages of drug design, the ADME/T properties of a drug are evaluated [35]. Absorption is the process of drug transport into the human circulatory system. Distribution is the penetration of the drug through the cell membrane barrier into the various tissues, organs or body fluids. As metabolism occurs, the initial (parent) compound is converted to new compounds called metabolites. The majority of small-molecule drug metabolism is carried out in the liver by redox enzymes, termed cytochrome P450 enzymes. Excretion is the removal of the initial form and metabolites of the drug from the human body. The toxicity of the drug also affects the human body. Several commercial software programs have equipped the ADME/T prediction module for drug molecules. For instance, the commercial software Discovery Studio provides an ADME/T descriptor module for candidate drug screening, which contains the following aspects: aqueous solubility, to predict the solubility of each compound in aqueous solvent; blood brain barrier penetration, to predict the ability of compounds to penetrate into the brain; and CYP2D6 enzyme binding capacity. The CYP2D6 enzyme is an important member of the CYP450 enzyme family and participates with the CYP3A4, CYP1A2, CYP2C9, and CYP2C19 enzymes in drug metabolism. The five major CYP enzymes are responsible for more than 95% of the drug metabolism in animals. The CYP2D6 enzyme normally accounts for approximately 2% of the CYP total but approximately 30% of the total drug metabolism. In clinical practice, the high CYP2D6 enzyme binding capacity of drug can reflect its excellent metabolism ability in the human body. The relevant criteria involve hepatotoxicity, the dose-dependent liver toxicity of the drug molecules; intestinal absorption, the absorption of drug molecules in the human body after oral administration; and plasma protein binding, the ability of the compound to bind to the carrier protein in the bloodstream.

Suitable pharmacokinetic properties and low toxicity during body absorption, distribution, metabolism and excretion are the key factors in successfully passing the clinical trials. Predicting the ADME/T properties of drug molecules prior to drug design and performing this screening can reduce the cost of drug development and improve the success rate of the whole procedure. For ADME/T properties rational predictive have been developed on mechanistic descriptions of the underlying biophysical processes. Some softwares can performing ADME/T such as Simulations Plus ADME/T predictor [36], PK-Map the Discovery Studio from Accerlary company [37].

#### Exclusion of false-positive compounds

Due to accidents or other reasons, some false-positive compounds are also included in the filtered sample library for the post-screening step. Most of these false-positive compounds are easily decomposed under hydrolytic conditions and react with proteins or biological nucleophiles (glutathione, dithiothreitol, etc.), which are easily detected by a positive result in an enzyme system or cell test. In fact, these false-positive compounds are mostly chemical reaction intermediates such as epoxides, aldehydes, haloalkanes, or compounds consisting of a conjugated system.

### The concept and performance of virtual screening

**Fig. 2:**
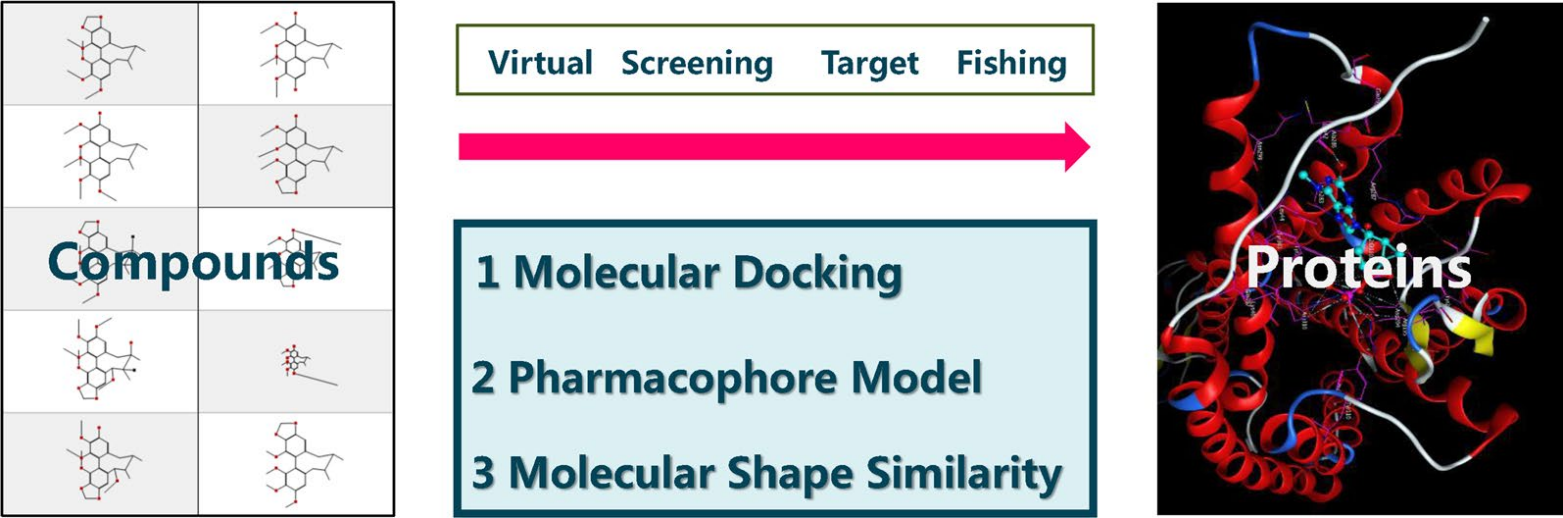
Demonstration of virtual screening methods

The virtual screening of drugs can be defined as follows: based on the theories of drug design and new drug screening, with the aid of computer technology and professional software, selecting the theoretically active lead compounds from large quantities of compounds and then evaluating the activity experimentally. Virtual screening methods have three main theoretical foundations: molecular docking, pharmacophore theory, and small molecular shape similarity. According to the author’s work experiences: these three methods have different applications and their own advantages and disadvantages (Table 2, Fig. 2).

**Table 2:** Three virtual screening methods comparison

Methods	Molecular docking [38]	Pharmacophore model [39]	Small molecule shape similarity [40]
Theory basis	Molecular mechanics, quantum mechanics	Statistics	Graph theory and other mathematical methods
Overview	Obtain the receptor structure information and locate its binding site, mimic the interaction between the receptor and its ligands	Establish pharmacophore model, evaluate the matching degree between ligands 3D conformation and pharmacophore models	To investigate the structural similarity of unknown molecules by druggable molecules at known targets
Advantages	1. Algorithm is mature 2. A variety of optional softwares	1. High accuracy and efficiency 2. Several commercial pharmacophore databases	1. Fastest screening 2. Abundant data resources
Disadvantages	1. Relatively large amount of calculation 2. Huge data preparation workload 3. Results analysis takes a long time	1. Affected by the quality of pharmacophore model 2. Affected by the amount of protein crystals	1. Low accuracy and rough results 2. Require operator able to develop chemical software

Molecular docking relies on the characteristics of the receptor (drug target/protein/enzyme) itself and the interaction pattern with its small molecule ligand to perform drug design based on the ligand-receptor binding mode (the lock and key principle) [41]. The electrostatic interactions, hydrogen bonding, hydrophobic interaction and van der Waals forces are calculated to predict the ligand’s binding mode and affinity. The pharmacophore is an abstract set of molecular features that are necessary for the molecular recognition of a ligand by a biological macromolecule [42]. The pharmacophore group refers to the spatial arrangement of the pharmacological characteristics of the small molecule in the drug, including the hydrogen bond donors, hydrogen bond acceptors, positive and negative charge centres, aromatic ring centre, hydrophobic groups, hydrophilic groups and geometric conformation. The biological activity of small molecules can be predicted by the summary of their pharmacological feature elements. Small molecule shape similarity can be defined as a database search technique based on the quantitative structure–activity relationships of compounds with the same mechanisms [43]. These three types of common screening software and their characteristics are listed in Table 3.

**Table 3:** Common virtual screening softwares overview

Softwares	Websites	Features	References
Molecular docking			
Affinity	http://www.accelrys.com/insight/affinity	Based on simulated annealing, molecular mechanics and molecular dynamics simulation of molecular pairs of procedures, the calculation is more accurate	[44]
AutoDock	http://www.scripps.edu/pub/olson-web/doc/autodock	Famous molecular docking program developed by the Scripps Institute. Which is one of the most widely used docking software	[44]
DOCK	http://www.dock.compbio.ucsf.edu	One of the most widely used molecular docking programs, free open access	[45]
DockVision	http://www.dockvision.com	A set of docking applets that support multiple algorithms	[46]
DockIt	http://www.metaphorics.com/dockit	Provide Energy, PLP and PMF evaluation methods	[47]
eHiTS	http://www.simbiosys.ca/ehits	eHiTS is an exhaustive flexible-docking method that systematically covers the part of the conformational and positional search space that avoids severe steric clashes, producing highly accurate docking poses at a speed practical for virtual high-throughput screening	[48]
FlexX	http://www.biosolveit.de/FlexX	The method can be used in the design process of specific protein ligands. It combines an appropriate model of the physico-chemical properties of the docked molecules with efficient methods for sampling the conformational space of the ligand	[49]
Glide	http://www.schrodinger.com/prods/glide	Glide approximates a complete systematic search of the conformational, orientational, and positional space of the docked ligand. In this search, an initial rough positioning and scoring phase that dramatically narrows the search space is followed by torsionally flexible energy optimization on an OPLS-AA nonbonded potential grid for a few hundred surviving candidate poses	[50]
GoldDock	http://www.ccdc.cam.ac.uk/prods/gold	GOLD is an automated ligand docking program that uses a genetic algorithm to explore the full range of ligand conformational flexibility with partial flexibility of the protein, and satisfies the fundamental requirement that the ligand must displace loosely bound water on binding. Numerous enhancements and modifications have been applied to the original technique resulting in a substantial increase in the reliability and the applicability of the algorithm	[51]
SystemsDock	http://www.systemsdock.unit.oist.jp	SystemsDock is a web server for network pharmacology-based prediction and analysis, which applies high-precision docking simulation and molecular pathway map to comprehensively characterize the ligand selectivity and to illustrate how a ligand acts on a complex molecular network	[52]
ZDOCK	http://www.zlab.bu.edu/rong/dock	Protein–Protein docking procedure based on geometric matching. With a goal of providing an accessible and intuitive interface, ZDOCK provide options for users to guide the scoring and the selection of output models, in addition to dynamic visualization of input structures and output docking models	[53]
Pharmacophore model			
Apex-3D	http://www.biosym.com/apex-3d	Activity prediction expert system with 3D-QSAR. pharmacophore identification based on logical structure analysis	[54]
DISCOtech	http://www.tripos.com/discotech	Distance comparison technique provide multi-drug group model for database search, auto recognize the priority	[55]
Discovery Studio	http://www.accelrys.com/	Powerful pharmacophore identification and database search software	[56]
GASP	http://www.tripos.com/gasp	Based on genetic algorithm to realize flexible stacking between drug molecules	[57]
SEAware	http://www.seachangepharma.com/store/academic/products	Chemically similar drugs often bind to biologically diverse targets, making it difficult to predict what off-target effects a drug might have by protein structure or sequence alone. The similarity ensemble approach (SEA) addresses this problem using a different strategy; it groups receptors according to the chemical similarity of their ligands, and can identify unknown relationships between ligands and receptors amenable to experimental testing	[58]
Small molecule shape similarity			
CerberuS		CerBeruS is a method developed for iterative screening. CerBeruS is based on Daylight fingerprints. CerBeruS proposes only highly similar molecules for testing. This strategy results in a high hit rate but is unlikely to identify new scaffolds or lead series	[59]
FlexS	http://www.biosolveit.de/FlexS/	FlexS is an incremental construction procedure. The molecules to be superimposed are partitioned into fragments. Starting with placements of a selected anchor fragment, computed by two alternative approaches, the remaining fragments are added iteratively. At each step, flexibility is considered by allowing the respective added fragment to adopt a discrete set of conformations. The mean computing time per test case is about 1:30 min on a common-day workstation	[60]
BRUTUS		BRUTUS aligns molecules using field information derived from charge distributions and van der Waals shapes of the compounds. Molecules can have similar biological properties if their charge distributions and shapes are similar, even though they have different chemical structures; that is, BRUTUS can identify compounds possessing similar properties, regardless of their structures	[61]
WEGA	http://www.rcdd.org.cn/home/program.html	(WEGA), is proposed to improve the accuracy of the first order approximation. The new approach significantly improves the accuracy of molecular volumes and reduces the error of shape similarity calculations by 37% using the hard-sphere model as the reference. The new algorithm also keeps the simplicity and efficiency of the FOGA (First Order Gaussian Approximation)	[62]

The focus of our research is to find the pharmacological basis of the effects of the constituents of medicinal plants. From this perspective, the pharmacophore model can be used to screen the large quantities of compounds from medicinal plants. In contrast, from the perspective of diseases and proteins, it is possible to use molecular docking to find the most likely active constituents.

Therefore, we design this core section methodology as follows: first, we perform batch screening based on the pharmacophore model theory to obtain the potential set of binding drugs. Subsequently, these results are verified by use of the small molecule shape similarity theory. Finally, when the target protein requires detailed study, molecular docking can be used to refine the active small molecules.

#### Virtual screening based on pharmacophore model

The geometrical matching and energy matching process between a drug molecule and its receptor are necessary features for the binding interaction. Different group structures exert different effects on the activity, while similar chemical characteristics have the same or similar pharmacological activities. The concept of the pharmacophore was first described by Paul Ehrlich in 1909 [63]. The pharmacophore refers to the molecular framework of atoms with active and essential features that allow identification of their pharmacological activities. In 1977, Peter Gund further defined the pharmacophore as a group of molecular features that participate in a molecular biological activity. At present, the definition of the pharmacophore is pharmacodynamics elements and their spatial arrangement. These ‘potency elements’ can be specific atoms such as an oxygen atom or carbonyl group or abstract functional structures: hydrogen bond acceptor/donor, positive/negative ionizable charge, aromatic ring, hydrophilic group, the location and orientation of atoms, or the distance, tolerance and exclusion volume of atoms. The pharmacophore is a summary of a series of biologically active molecules that effectively describes the structural features that play the most important roles in activity. The pharmacophore is not representative of a single molecule or a specific functional group. It can represent a group of compounds and the important interaction information on the same receptor class or can be considered a large number of active compounds with common drug characteristics. The construction of a pharmacophore model can be used for the virtual screening of a small molecule library, searching for new skeletal active molecules, predicting the activity of compounds, and optimizing and modifying them. The pharmacophore can also qualitatively quantify the structure–activity relationships of compounds and elucidate the selectivity mechanism of the compounds [64]. During the process of virtual screening, the pharmacophore model can be used to characterize the active conformation of the ligand molecule by conformational search and molecular superposition, and the possible mode of action between the receptor and the ligand molecule can be deduced and explained accordingly.

In addition, a reverse search based on the pharmacophore can solve several common issues focused on the chemical constituents of the medicinal plants, such as its specific targets, therapeutic effect and how to find or construct a similar role among synthesized compounds. Currently, the active ingredients of the medicinal plans can be elucidated by utilizing different commercial software launched by many companies, which can afford virtual screening modules based on pharmacophore theory. We recommend The Discovery Studio developed by the Accelary Company, which equipped the PharmaDB and Hypo DB, these databases contained thousands of pharmacophore models from sc-PDB recorded information.

#### Validation based on ligand molecule shape similarity

Molecular shape similarity analysis is based on the similarity of small molecules as the index for the database mapping search. Similar to pharmacophore theory, molecular shape similarity analysis can effectively utilize the overall structural characteristics of those compounds. For now, molecular shape similarity analysis is specifically applied to scaffold hopping and shape discrimination [65].

Due to the rule that “molecules with similar structure may have similar or the same biological activity”, during the drug design process, a pharmacist might choose the ligand shape similarity screening method. Generally, small molecule compounds with known activity and targets are used to search for the molecular structure of chemicals in the compound database according to the shape similarity of the potential sample compounds.

During the establishment of this methodology, since the molecular shape similarity screening approach involves a faster process but more challenging result limitations and peculiarities, this method is not suitable for use as the main approach in virtual screening but rather as a validation based on efficacy. At present, many commercial software programs can perform molecular shape similarity operations, including CerberuS, FlexS, and MIMIC. We suggest to choose the WEGA (weighted Gaussian algorithm), is a typical virtual screening method based on ligand shape similarity that was established by Professor Xu Jun’s group from Sun Yat-sen University [56]. The WEGA already can performing in TianHe No. 2 supercomputing platform to ensure its efficiency. The accuracy of the results from another pathway can be evaluated by comparing the shape similarity between the small molecules of those ligands corresponding to the known drug targets and the small molecules yet to be tested.

#### Accurate verification based on molecular docking theory

The conceptual basis of molecular docking originated from the “lock and key principle model” proposed 100 years ago. The principle of molecular docking is to place a series of test compounds with known three-dimensional structures in turn at the active site of the biological target molecule [66]. The optimal binding conformation of the compound with the target molecule is found by continuously improving the conformation, position, dihedral angle of each rotatable bond, and amino acid residue side chain and backbone of the compound in space and predicting their combination patterns and affinities. The molecular docking method can use a variety of scoring standards to select the most natural conformation of the compound and optimize its affinity to the target according to theoretical analogue molecular interactions. Autodock, FlexX, ICM, GoldDocK, Ligand-Fit and Glide can all perform molecular docking. Due to the accuracy, sensitivity and specificity of the molecular docking, we can use it to refine the results obtained from the pharmacophore model and shape similarity theory. The Autodock is the most used tool for molecular docking in recently years due to its fast and friendly interface. We also recommend LigandFit algorithm: A method employs a cavity detection algorithm for detecting invaginations in the protein as candidate active site regions. A shape comparison filter is combined with a Monte Carlo conformational search for generating ligand poses consistent with the active site shape. Candidate poses are minimized in the context of the active site using a grid-based method for evaluating protein–ligand interaction energies and due to its can comparing enormous poses of ligands, which is suitable for high-throughput screening.

### Analysis of target sets

#### Analysis and annotation of target information

After obtaining the druggable targets by virtual screening, we need to analyse the relevant information on the targets: target type, protein structure, binding pocket shape, associated pathway and corresponding diseases. The targets are usually nominated according to their UniProt ID. UniProt is an abbreviation for the Universal Protein Database program, which consists of the Swiss-Prot, TrEMBL and PIR-PSD databases and is the largest database containing the most informative data resources and protein structures [67]. Using the UniProt ID, detailed information regarding the target protein can obtained at the UniProt website (http://www.uniprot.org/), including the protein name, gene name, organism, sequence information, taxonomy, family and domains, and related molecular functions and biological processes. This database can also link to other databases by searching based on the PDB ID, KEGG ID and other information. In addition to UniProt, many other databases and software programs can also analyse the category and function of these fished targets and their corresponding diseases. Table 4 summarizes the commonly used databases and analysis software.

**Table 4:** Common druggable targets/protein databases overview

Databases	Developers	Websites	Abstract	References
Binding DB	Skaggs School of Pharmacy & Pharmaceutical Sciences, USA	http://www.bindingdb.org/bind/index.jsp	BindingDB is a public, web-accessible database of measured binding affinities, focusing chiefly on the interactions of proteins considered to be candidate drug-targets with ligands that are small, drug-like molecules	[68]
BioGRID (Biological General Repository for Interaction Datasets)	BioGRID, Canada	http://www.thebiogrid.org	BioGRID is an interaction repository with data compiled through comprehensive curation efforts. Our current index is version 3.4.151 and searches 63,354 publications for 1,493,749 protein and genetic interactions, 27,785 chemical associations and 38,559 post translational modifications from major model organism species. All data are freely provided via our search index and available for download in standardized formats	[69]
DAVID (Database for Annotation, Visualization, and Integrated Discovery)	Laboratory of Human Retrovirology and Immunoinformatics, USA	http://www.david.ncifcrf.gov	The database for annotation, visualization and integrated discovery Able to perform: Identify enriched biological themes, discover enriched functional-related gene groups, list interacting proteins and other functions	[70]
DRUGBANK	Canadian Institutes of Health Research, Canada	http://www.drugbank.ca	The DrugBank database is a unique bioinformatics and cheminformatics resource that combines detailed drug data with comprehensive drug target information. The database contains 9591 drug entries including 2037 FDA-approved small molecule drugs, 241 FDA-approved biotech drugs, 96 nutraceuticals and over 6000 experimental drugs. Additionally, 4661 non-redundant protein (sequences are linked to these drug entries	[71]
GeneMANIA	University of Toronto, Canada	http://www.genemania.org	GeneMANIA finds other genes that are related to a set of input genes, using a very large set of functional association data. Association data include protein and genetic interactions, pathways, co-expression, co-localization and protein domain similarity. You can use GeneMANIA to find new members of a pathway or complex, find additional genes you may have missed in your screen or find new genes with a specific function, such as protein kinases. Your question is defined by the set of genes you input	[72]
HPRD (Human Protein Reference Database)	Johns Hopkins University and the Institute of Bioinformatics, USA	http://www.hprd.org	HPRD is a database of curated proteomic information pertaining to human proteins. All the information in HPRD has been manually extracted from the literature by expert biologists who read, interpret and analyze the published data	[73]
IntAct	European Molecular Biology Laboratory	http://www.ebi.ac.uk/intact	IntAct is an open-source, open data molecular interaction database populated by data either curated from the literature or from direct data depositions	[74]
KEGG (Kyoto Encyclopedia of Genes and Genomes)	Kyoto University, Japan	http://www.kegg.jp	KEGG (Kyoto Encyclopedia of Genes and Genomes) is a database resource that integrates genomic, chemical and systemic functional information. In particular, gene catalogs from completely sequenced genomes are linked to higher-level systemic functions of the cell, the organism and the ecosystem. KEGG is widely used as a reference knowledge base for integration and interpretation of large-scale datasets generated by genome sequencing and other high-throughput experimental technologies. In addition to maintaining the aspects to support basic research, KEGG is being expanded towards more practical applications integrating human diseases, drugs and other health-related substances	[75]
MAS3.0	CapitalBio, China	http://www.bioinfo.capitalbio.com	MAS (Molecule Annotation System) is a whole data-mining and function annotation solution to extract and analyze biological molecules relationships from public knowledgebase of biological molecules and signification. MAS analysis platform is a web client program for interactive navigation in the knowledge base. MAS uses relational database of biological networks created from millions of individually modeled relationships between genes, proteins, diseases and tissues. MAS allow a view on your data, integrated in biological networks according to different biological context. This unique feature results from multiple lines of evidence which are integrated in MAS’ database. MAS Help to understand relationship of gene expression data	[76]
MINT (The Molecular INTeraction Database)	Department of Biology, University of Rome, Italy	http://www.mint.bio.uniroma2.it/	The MINT is a public repository for protein–protein interactions (PPI) reported in peer-reviewed journals. The database grows steadily over the years and at September 2011 contains approximately 235 000 binary interactions captured from over 4750 publications	[77]
PharmMapper Server	Shanghai Institute of Materia Medica, China	http://lilab.ecust.edu.cn/pharmmapper/index.php	PharmMapper Server is a freely accessed web-server designed to identify potential target candidates for the given probe small molecules (drugs, natural products, or other newly discovered compounds with binding targets unidentified) using pharmacophore mapping approach. Benefited from the highly efficient and robust mapping method, PharmMapper bears high throughput ability and can identify the potential target candidates from the database within a few hours	[78]
Potential Drug Target Database (PDTD)	Shanghai Institute of Materia Medica, China	http://www.dddc.ac.cn/pdtd/index.php	PDTD is a dual function database that associates an informatics database to a structural database of known and potential drug targets. PDTD is a comprehensive, web-accessible database of drug targets, and focuses on those drug targets with known 3D-structures. PDTD contains 1207 entries covering 841 known and potential drug targets with structures from the Protein Data Bank	[79]
RCSB PDB (Protein Data Bank)	Research Collaboratory for Structural Bioinformatics: Rutgers and UCSD/SDSC	http://www.rcsb.org/pdb/home	A global resource for the advancement of research and education in biology and medicine. Along with our Worldwide PDB collaborators, RCSB PDB curates, annotates, and makes publicly available the PDB data deposited by scientists around the globe. The RCSB PDB then provides a window to these data through a rich online resource with powerful searching, reporting, and visualization tools for researchers	[80]
Reactome	European Bioinformatics Institute (EMBL-EBI)	http://www.reactome.org	The Reactome provides molecular details of signal transduction, transport, DNA replication, metabolism and other cellular processes as an ordered network of molecular transformations—an extended version of a classic metabolic map, in a single consistent data model	[81]
STITCH (search tool for interactions of chemicals)	European Molecular Biology Laboratory, Germany	http://www.stitch.embl.de	STITCH is a database of known and predicted interactions between chemicals and proteins. The interactions include direct (physical) and indirect (functional) associations; they stem from computational prediction, from knowledge transfer between organisms, and from interactions aggregated from other (primary) databases	[82]
STRING (Search Tool for the Retrieval of Interacting Genes/Proteins)	Swiss Institute of Bioinformatics, Switzerland	http://www.string-db.org	STRING is a database of known and predicted protein–protein interactions. The interactions include direct (physical) and indirect (functional) associations; they stem from computational prediction, from knowledge transfer between organisms, and from interactions aggregated from other (primary) databases	[83]
TTD (Therapeutic Target Database)	Department of Computational Science National University of Singapore, Singapore	http://www.bidd.nus.edu.sg/group/cjttd	Therapeutic target database (TTD) is a database to provide information about the known and explored therapeutic protein and nucleic acid targets, the targeted disease, pathway information and the corresponding drugs directed at each of these targets. Also included in this database are links to relevant databases containing information about target function, sequence, 3D structure, ligand binding properties, enzyme nomenclature and drug structure, therapeutic class, clinical development status. All information provided are fully referenced	[84]
Uniprot (Univeral Protein)	UniProt Consortium	http://www.uniprot.org	The Universal Protein Resource (UniProt) is a comprehensive resource for protein sequence and annotation data. The UniProt databases are the UniProt Knowledgebase (UniProtKB), the UniProt Reference Clusters (UniRef), and the UniProt Archive (UniParc)	[67]

#### Construction of network pharmacology

In contrast to the chemical drug action model, traditional medicinal herbs, TCMs or formulas all have a wide variety of ingredients, a broad range of drug targets and complex mechanistic characteristics, leading to challenges in elucidating their mechanisms and action models. Network pharmacology is based on the theory of systems biology. It treats each drug, target, gene, pathway and disease as a specific signal node and each action model as an edge to construct a topological network map of their complex relationships [85]. The multi-component, multi-target and multi-pathway mechanisms of medicinal plants can be elucidated by analysing the interrelationships in this topological network map, making traditional medicinal plants available for modern research and innovation. A variety of software programs and web databases can currently analyse the relevant information on target sets and their related pathways and diseases. Examples include the Ingenuity Pathway Analysis (IPA) software from QIAGEN Bioinformatics [86], the KEGG pathway database developed by Kyoto University [87] and MetaCore by Thomson Reuters [88]. The analyses of the principles and characteristics performed by each program also vary. The Causal Network Analysis and Upstream Regulator Analysis tools in IPA can predict the type of disease and associated signalling pathways that correspond to a list of genes and give separate weights for the resulting compound data set. MetaCore uses a massive collection of literature information to identify the small molecules and related proteins corresponding to the underlying disease pathways.

Through the above steps, we can obtain a large amount of information on a medicinal plant, compound, target, pathway, or disease and its interrelationships by analysing the results of the virtual screening and target analysis. Different network visualization tools can show the relationships between these related nodes. Commonly applied software programs and their features are summarized in Table 5. Among them, the open access software Cytoscape is currently the most widely used tool due to its powerful graphical effects and its extensive compatibility with other software programs and databases. The core function of Cytoscape is network construction, which can build a two-dimensional topological network map within each node by edges and then concisely and clearly analyse the pharmacological basis and mechanism of medicinal plants. Each type of node (protein, compound and disease) and their relationship strength can be edited and analysed separately. Cytoscape can also link directly to external public databases and currently offers a variety of plugins to satisfy diverse analysis requirements. Pajek means spider in Slovenian and is another software program for network analysis. Pajek has the ability to break a large and complex network into smaller networks to utilize more efficient methods for further processing. It can also supply powerful visualization tools to implement large-scale (subquadratic) network algorithm analysis.

**Table 5:** Common visualization tool overview

Tools	Websites	Features	References
CADLIVE	http://www.cadlive.jp	CADLIVE (Computer-Aided Design of LIVing systEms) is a comprehensive computational tool for constructing large-scale biological network maps, analyzing the topological features of them, and simulating their dynamics	[89]
Cytoscape	http://www.cytoscape.org	Cytoscape is an open source software platform for visualizing molecular interaction networks and biological pathways and integrating these networks with annotations, gene expression profiles and other state data	[90]
Graphviz	http://www.graphviz.org	Graphviz is open source graph visualization software. Graph visualization is a way of representing structural information as diagrams of abstract graphs and networks. It has important applications in networking, bioinformatics, software engineering, database and web design, machine learning, and in visual interfaces for other technical domains	[91]
Pajek	http://www.mrvar.fdv.unilj.si/pajek	Pajek is a program package for analysis and visualization of large networks (networks containing up to one billion of vertices, there is no limit-except the memory size-on the number of lines). It has been available for 20 years	[92]
VANTED	http://www.vanted.ipkgatersleben.de	VANTED is a tool for the visualization and analysis of networks with related experimental data. Data from large-scale biochemical experiments is uploaded into the software via a Microsoft Excel-based form. Then it can be mapped on a network that is either drawn with the tool itself, downloaded from the KEGG Pathway database, or imported using standard network exchange formats. Transcript, enzyme, and metabolite data can be presented in the context of their underlying networks	[93]
VisANT	http://www.visant.bu.edu/	VisANT is an application for integrating biomolecular interaction data into a cohesive, graphical interface. This software features a multi-tiered architecture for data flexibility, separating back-end modules for data retrieval from a front-end visualization and analysis package	[94]
YANAsquare	http://www.yana.bioapps.biozentrum.uni-wuerzburg.de	YANAsquare provides a software framework for rapid network assembly (flexible pathway browser with local or remote operation mode), network overview (visualization routine and YANAsquare editor) and network performance analysis (calculation of flux modes as well as target and robustness tests)	[95]

Vine tea (VT) originates from the plant *Ampelopsis grossedentata*, which belongs to the family Vitaceae. VT is mainly distributed in the south of China, and pharmacological studies have shown that it has anti-inflammatory and anti-tumour functions and can decrease hypoglycaemia and protect liver function [96]. However, its specific activity components and their mechanism remain unclear. Using the methodology constructed previously, its mechanism can be adequately predicted.

Firstly, we collected 94 compounds from VT which were all reported, and all chemical structures were prepared in SD format. Molecular energy was minimized using the Energy Minimization module of Discovery Studio version 4.5 (DS 4.5; Accelrys Inc., San Diego, CA, USA) under the chemistry at Harvard Macromolecular Mechanics (CHARMM) force field and construction of the 3D multi-conformational VT compounds molecular structure database (VT-DB), which was generated by a Monte Carlo-based conformational analysis (FAST mode). After ADME/T selection in DS4.5 ADME/T property module, the pharmacophore-based target fishing was performed in DS4.5. All those fished protein targets were through the second refine by using WEGA validation. For each target, the protein name, gene name, and pathway information were collected from the PDB, KEGG, DRUNGBANK and TDD. The overall VT compound-target-pathway networks were generated using Cytoscape V3.2.

**Fig. 3:**
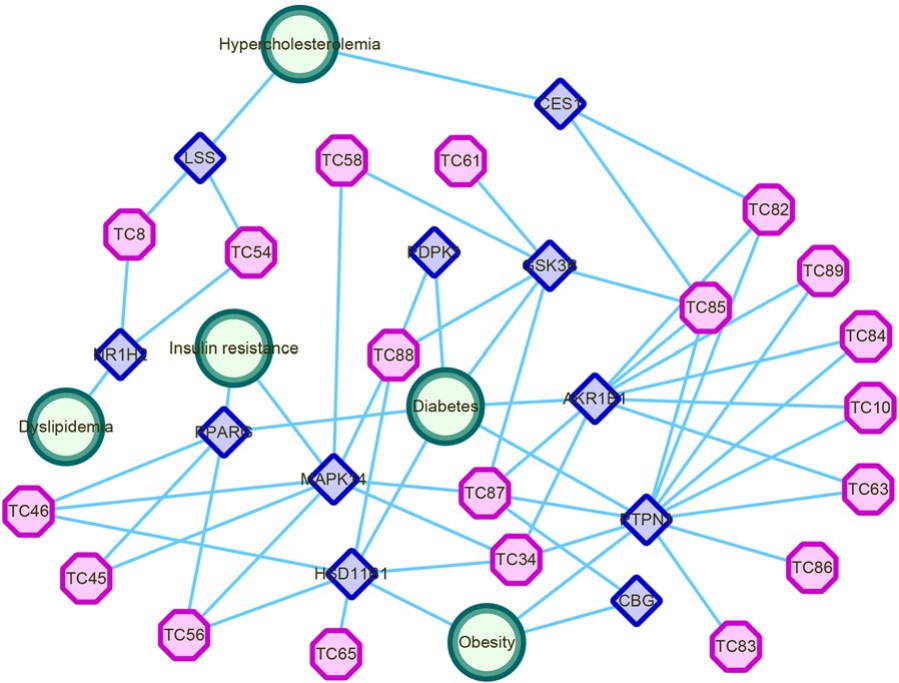
The topological network relationships between compounds, targets and diseases associated with VT

In Fig. 3, the topological analysis of the interaction network provides insights into its biologically relevant connectivity patterns. The pharmacological network of VT contains three types of nodes. The compounds extracted from VT form the external part of the network (pink octagon) and surround their fished target nodes (blue quadrangle). Each target is linked to at least one disease (green circle). A general overview of the global topological properties of the VT network can obtained from this figure. The figure clearly shows that the effects of the compounds in VT are usually “multiple to multiple”, which reflects the material basis of the VT and its hypoglycaemic and lipid-lowering activities.

## Conclusion and perspectives

In this paper, we have proposed a wide-ranging complete technical route that utilizes a series of in silico approaches to reveal the pharmacological basis of the effects of medicinal plants, first to determine the object of study and then to obtain its natural constituents and construct the compound database. After pre-processing of the included compounds, a three-step virtual screening is performed. Finally, network pharmacology tools can be used to analyse the target set obtained from the screening; sketch out the network topological map of the medicinal plants, natural constituents, biological targets, associated pathways, related diseases and their interactions; and elaborate on the results.

The proposed joint methodology combines multiple in silico approaches and, through the full use of a series of virtual screening and network pharmacology tools, can clarify the substance basis and the mechanism of medicinal plants. The first half of this methodology uses software technology, including compound activity forecasting and virtual screening, all based on mass experimental data, to provide pinpoint accuracy. This methodology is superior to conventional screening because it requires only structural information on compounds and can perform multi-target screening of a vast number of compounds in a relatively short period of time, which can drastically reduce the cost and time of drug development. Moreover, combining this in silico methodology with pertinent pharmacological experiments could significantly enhance the efficiency of medicinal plant research and new drug discovery. The latter half of the methodology is guided by network pharmacology theory and uses multiple analysis tools and related databases to obtain relevant information from massive bio-information resources; build a network topological map of medicinal plants, chemical constituents, genes, proteins, pathways and diseases; and perform data mining and analysis.

This in silico methodology can resolve the status of medicinal plants that are difficult to study on a practical level and can predict and clarify the mechanisms of the active ingredients in medicinal plants.

Although computer-aided drug design and network pharmacology have been widely used and developed, there still have deficiencies and limitations: (1) The model maturity and computational accuracy of computer docking algorithms need to be further improved. (2) Due to the structure-based methodology, several compounds are not suitable for computer-aided design because of their special structure characteristics. (3) A large number of databases can improve different information for the obtained potential targets, the progress of the selection of these databases and their effective information annotation, still requires continuously practical activities to optimize. With the advancement of computer science, and the constant optimization of algorithms, including the maturity of the protein model. Through more practical researches and development examples to upgrade the entire process of in silico methodology, we believe that in the future, this methodological process will enable the discovery of new drugs more efficiently, accurately and quickly. This methodology will be more widely usable in future work on revealing and predicting the basis of medicinal materials.

## Abbreviations

TCM: Traditional Chinese medicine
ADME/T: absorption, distribution, metabolism, excretion and toxicity
RO5: Lipinski’s rule of five
WEGA: weighted Gaussian algorithm
VT: vine tea
